# Supplementary material for: Drosophila Models Reveal NAT Complex Roles in Heart Development and Enable Functional Validation of Congenital Heart Disease Variants
Source: Cells. 2025 Oct 14;14(20):1596. doi: 10.3390/cells14201596 (PMC12564477; doi:10.3390/cells14201596)
Supplement: Supplementary file 1 [file cells-14-01596-s001.zip › cells-3899545-supplementary.pdf]

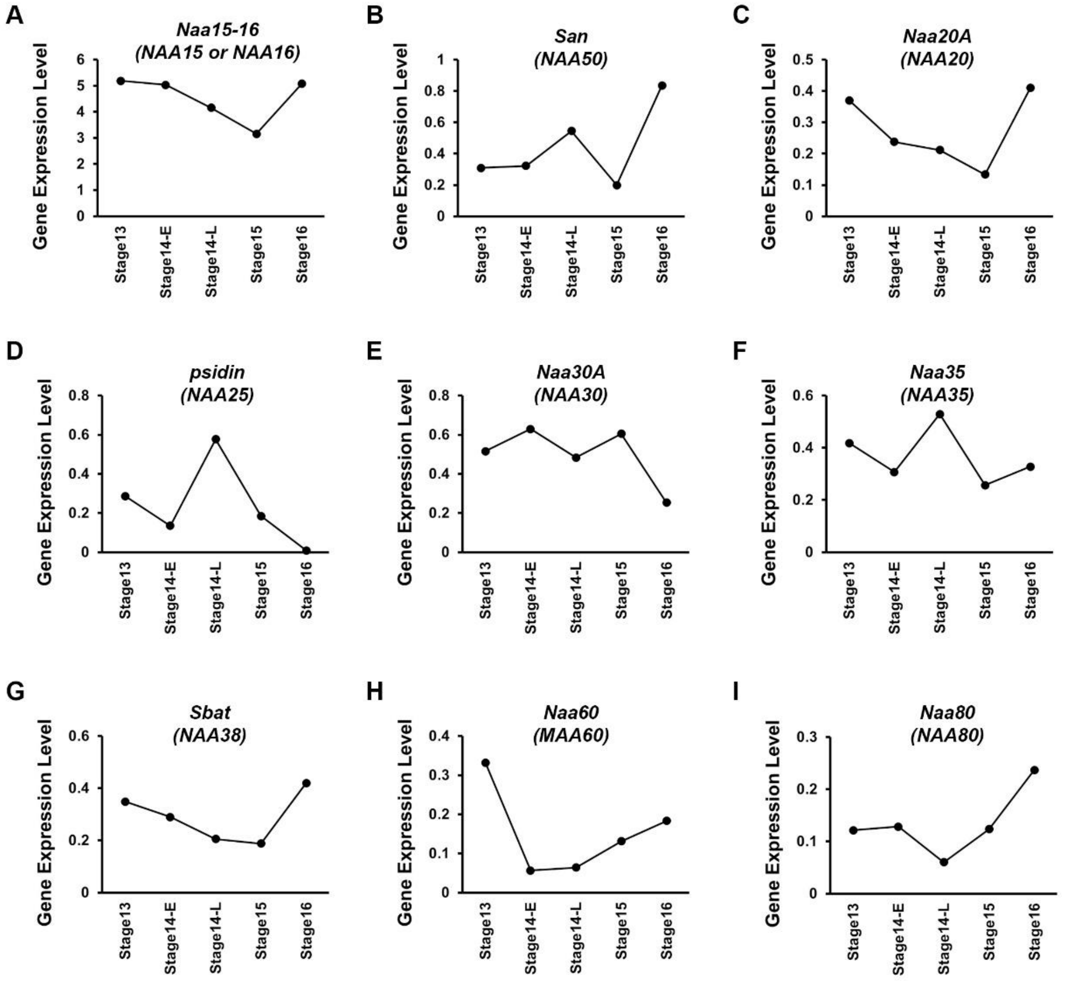

Supplemental Figure S1. Relative gene expression level for NAT complex components in *Drosophila* heart. The relative gene expression level for genes encoding NAT complex components in *Drosophila* cardiogenic progenitors at embryonic stages 13, 14-E (early), 14-L (late), 15, and 16. *vnc* expression was undetectable in cardiac progenitor cells at any of the examined stages. None of the changes reached statistical significance.

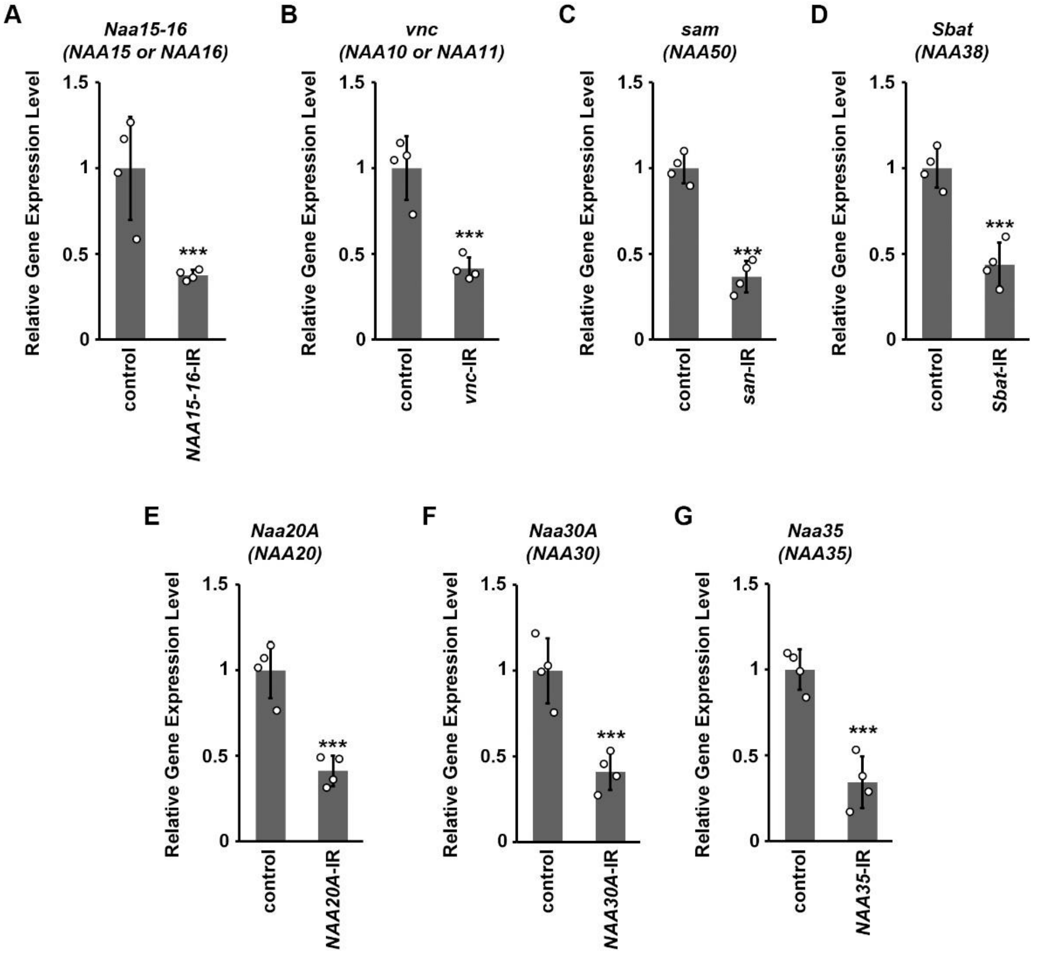

Supplemental Figure S2. Relative gene expression level after NAT complex components silenced in *Drosophila* heart. The relative gene expression level in adult heart (4-day-old females) following the expression of UAS-RNAi transgenes targeting genes encoding NAT complex components (4XHand-Gal4). [mean±s.d.; n=4 biological replicates per genotype, 30 flies per each sample (4-day-old females); Student's t-test; statistical significance: ***P<0.001].

Gene Name		
Gapdh	Forward	GAT TTG TTG TTG GCC GCA GT
	Reverse	GAA GTG GTTCGC CTG GAA GA
NAA15-16	Forward	GTCGATCTATCGCGTG
	Reverse	TACTTCGCGCACTTCG
vnc	Forward	CTTAGCTACGTGGCCGTTGA
	Reverse	GGACACGTACTGGGCATTGA
san	Forward	TGTCGCATCGACAACACTGA
	Reverse	GTCCTTCTCGGCGAAGTTCA
Sbat	Forward	ATCGTGATTACGGACGGACG
	Reverse	GCTCGTCGATGCTTAGGGAG
NAA20A	Forward	GAGGGACACCTGGACAACTG
	Reverse	ATGTCGTACGCATCCTCGTC
NAA30A	Forward	CACAACGACGGACCCAAAAC
	Reverse	GCATATCCAGCTTGCACACG
NAA35	Forward	GAACCAACTGGCCAACGAAC
	Reverse	GATCAGCATCGCACGGAAAC

Supplemental Table S1. Primer sequences used for quantitative RT-PCR analysis.
